# Structural Design and Performance of a Low-Frequency Hybrid Vibration Energy Harvester Based on Piezoelectric–Electromagnetic–Triboelectric Coupling

**DOI:** 10.3390/mi17030280

**Published:** 2026-02-25

**Authors:** Xingtong Chen, Yufan Zhu, Yuxuan Sheng, Xuan Ma

**Affiliations:** 1 Southampton Ocean Engineering Joint Institute at HEU, Harbin Engineering University, Harbin 150001, China; 2 College of Power and Energy Engineering, Harbin Engineering University, Harbin 150001, China

**Keywords:** vibration energy harvester, spiral cantilever beam, hybrid energy conversion, piezoelectric, electromagnetic, triboelectric

## Abstract

This study investigates a low-frequency piezoelectric–electromagnetic–triboelectric hybrid vibration energy harvester designed to address the narrow operating bandwidth of conventional vibration energy harvesters. The integrated design comprises a piezoelectric-electromagnetic generator module based on a spiral cantilever beam and a triboelectric nanogenerator module, with the objective of capturing and amplifying energy generated through both resonant and stochastic vibrations. Theoretical frameworks and simulations, conducted using COMSOL Multiphysics software, are used to analyze key design parameters and device performance. The physical fabrication involves advanced manufacturing techniques such as 3D printing and CNC machining. Subsequent experimental testing validates the success of the hybrid approach, achieving a maximum averaged output power of 2.86 mW and a maximum energy conversion efficiency of 36.81%. These findings underscore the feasibility and efficacy of this study in expanding the frequency domain and enhancing power generation capacity.

## 1. Introduction

In recent years, vibration and noise energy capture has been widely studied as a clean energy-harvesting approach with high practical feasibility and technological maturity, which uses the mechanical vibration of the environment to generate electricity, effectively making up for the limited availability of existing energy, labor constraints and waste pollution, and has great potential in coping with the global energy crisis [1]. At present, there are many vibrational energy harvester (VEH) devices on the market, most of which focus on capturing two forms of vibration, mechanical resonance and random vibration, and are designed based on a specific energy conversion mechanism, including electromagnetic [2,3,4], piezoelectric [5,6,7], triboelectric [8,9,10], and electrostatic [11,12,13] power generation methods. Each power generation method has its own advantages and disadvantages, and researchers have designed and developed various energy capture devices according to different needs. The principle of piezoelectric power generation operates via the piezoelectric effect. Upon the application of stress to piezoelectric materials, charges are induced within the material, subsequently generating voltage and current, thereby facilitating the transformation of energy. Piezoelectric power generation has a high output voltage and promising environmental adaptability, but it has a high output impedance, and the material is easy to fatigue. The most prevalent structure for a piezoelectric vibration energy harvester is the cantilever beam configuration. By appending a mass block to the extremity of the beam, it enables the piezoelectric material to attain elevated stress and output power at a comparatively lower resonance frequency. For this, the mirrored rotating p-VEHs proposed by Gang Yu et al. represents a remarkable advancement, capable of directly supplying power to a substantial number of Light-Emitting Diodes (LEDs) at optimal power levels [14]. Electromagnetic power generation can collect high-frequency vibration energy to output a large current, but the disadvantages are that the magnet and coil take up a large amount of space, and integrating the device is difficult. Based on this, Imbaquingo, C. et al.’s design of an elliptical electromagnetic vibration energy harvesting device stands out as a testament to the adaptability of these technologies. Its applicability to both one-dimensional and two-dimensional motions, coupled with ease of resonance frequency tuning, showcases the versatility required for practical implementation [15]. The operational principle of the Triboelectric Nanogenerator (TENG) is predicated on contact electrification and electrostatic induction. It capitalizes on the friction or contact between two disparate materials to instigate charge separation. Subsequently, these charges are transmuted into electrical energy via an external circuit. TENG has superior performance in harvesting small-scale mechanical energy across a wide frequency range, attracting significant attention due to its high performance, widespread availability, and cost-effectiveness. However, stable operation of TENGs requires well-controlled material properties and consistent interfacial contact conditions. The triboelectric vibration energy harvester developed by Kumar, S. et al. not only holds promise for widespread usage but also introduces the potential for significant advancements in self-powered electronic devices and systems [16]. The operational principle of electrostatic power generation is predicated on electrostatic induction and the energy transformation of the electrostatic field, thereby culminating in the formation of high-voltage static electricity. An electrostatic generator uses the electrostatic effect to generate electricity, which has the characteristics of high output power and wide collection frequency range, but this technology is not reflected in most VEH devices due to the requirement of external voltage support and excitation. In the domain of electrostatic vibration energy harvesters, Takhedmit, H. et al.’s utilization of a 2.4 GHz Cockcroft–Walton rectifying antenna for wireless pre-charging exemplifies creative approaches to enhancing power generation efficiency. This strategic implementation has resulted in commendable outcomes, emphasizing the adaptability and effectiveness of such technologies in practical applications [17].

In recent years, significant progress has been made in hybrid vibration energy harvesters in the quest for more efficient and versatile technologies, resulting in breakthroughs in composite power generation. As the name suggests, this kind of process involves the integration of two or more of the aforementioned power generation modes. Composite power generation is not merely a simplistic assembly of various power generation modules. Instead, it capitalizes on the common requirements of device structure and motion characteristics across different power generation methods to organically integrate multiple power generation modules. This integration approach does not impede the normal operation of each power generation module, while also effectively utilizing space, thereby enhancing power generation efficiency and space utilization rate. In comparison to the singular vibrational energy capture power generation mode, this approach exhibits a broader range of applicable operating conditions, effectively addressing the technical gaps inherent in singular power generation. Given this backdrop, a substantial cohort of researchers are dedicated to the development of composite vibration energy harvesters [18,19,20]. Furthermore, Yang, C. et al. devised a VEH based on piezoelectric and electrostatic effects. If this structure is implemented at the MEMS level at a small size, the novel hybrid piezoelectric and electrostatic VEH is anticipated to achieve a maximum power output of approximately 100 μW [21,22]. Additionally, Yang, X. et al. proposed a spring-assisted multi-stable nonlinear vibration energy harvesting technique, which, employing electromagnetic and frictional power generation principles, enables the utilization of more vibrational energy under random and low-amplitude excitation conditions [23]. Magnetic-force-based parameter tuning has also been demonstrated to effectively adjust the dynamic response of piezoelectric–electromagnetic hybrid harvesters [24]. Works of this nature, undertaken by these researchers, have propelled the advancement of the vibration energy harvester (VEH) field, achieving synergies among various power generation modes.

However, the main challenges faced by most vibration energy harvesting devices currently lie in the limitations of power generation and the effective working frequency domain. On one hand, vibration energy, as a low-quality mechanical energy that is difficult to collect, is mostly collected through the mechanical vibrations of the device when recovering this energy. This leads to a smaller amount of vibration energy that can be collected, lower energy recovery efficiency, and lower power output of the vibration power generation device. On the other hand, due to the limitations of the energy collection structure, the effective working frequency of most vibration energy harvesting devices is near their own natural frequency. It is necessary to broaden their effective energy collection frequency domain so that they can still effectively recover vibration energy when the vibration frequency of the working environment changes, thereby improving the universality of the vibration energy harvesting device. Vibration energy harvesting devices that combine multiple power generation methods have become a feasible solution that can simultaneously improve power output and broaden the effective working frequency domain through specific structural design, and they have the potential to revolutionize the efficiency and versatility of vibration energy harvesting technology.

Among hybrid energy conversion mechanisms, triboelectric nanogenerators are of particular interest from a tribological perspective, as their electrical output originates from contact electrification and interface-related charge transfer between dissimilar materials.

In this paper, a novel low-frequency piezoelectric–electromagnetic–triboelectric hybrid vibration energy harvester (PET-HVEH) is designed and examined. The piezoelectric–electromagnetic generator (P-EMG) module based on the spiral cantilever beam utilizes the structure of piezoelectric ceramics and magnetic coil. Through the piezoelectric effect and electromagnetic induction, it converts the mechanical energy generated by the forced vibration transmitted by the spring and the spiral cantilever beam into electrical energy. The uniquely designed spiral cantilever beam structure can effectively broaden the working frequency domain of the device. The triboelectric generator (TENG) module consists of four identical vertical contact-discrete triboelectric nanogenerators, which collect vibrational energy by the contact separation of polyamide (PA66) and fluorinated ethylene propylene (FEP) dielectric triboelectric films. After establishing and analyzing the theoretical model, the finite element analysis software COMSOL Multiphysics (version 5.0, COMSOL AB, Stockholm, Sweden, accessed on 9 February 2026) was used to simulate the mechanical properties of the vibrating structure, and data collection and analysis were carried out through product laboratory tests and tests in real physical environments. Through a series of research, the energy harvesting device has shown good results in collecting vibration energy, effectively improving the power output and broadening the effective working vibration frequency range of the device. This substantiates the feasibility of integrating this device into practical production applications, affirming the promising developmental prospects of vibration noise harvesting technology. Based on the above review of existing vibration energy harvesting technologies.

The main contributions of this work are summarized as follows:

(1) A spiral cantilever beam configuration is proposed to achieve low-frequency resonance within a compact structural volume.

(2) A hybrid piezoelectric–electromagnetic–triboelectric (PET) architecture is designed to capture both resonant and non-resonant vibration energy.

(3) Systematic experiments are conducted to validate bandwidth broadening and enhanced output power under low-frequency vibration excitation.

## 2. Design and Working Principle

### 2.1. Design Concept

**Figure 1:**
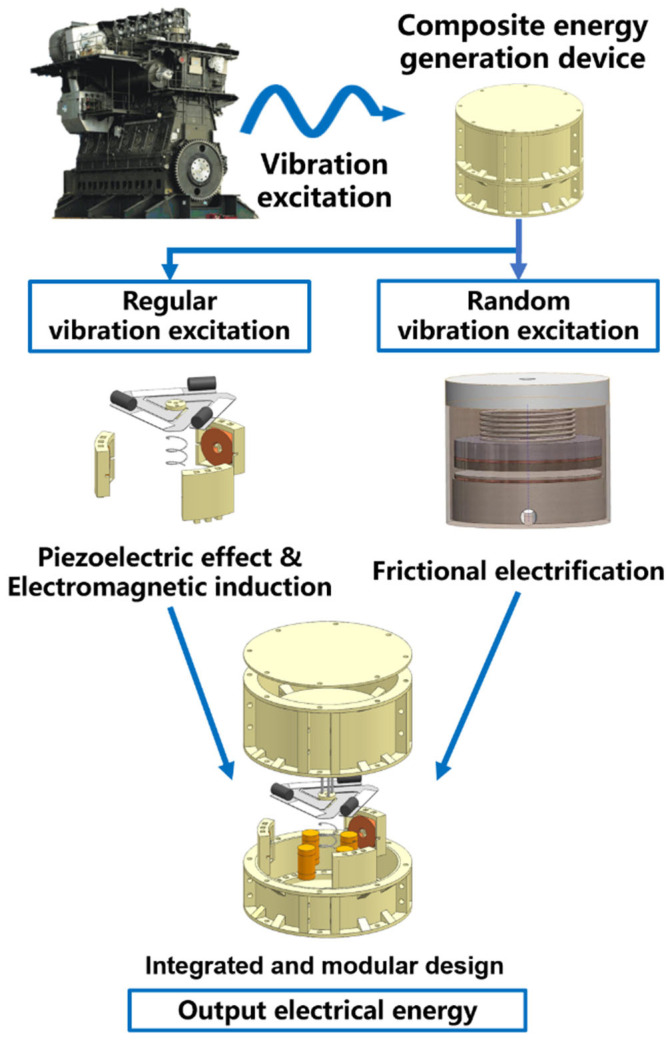
Flow chart of device design ideas.

The design concept of PET-HVEH is illustrated in Figure 1. The concept of combined vibration energy harvesting is present throughout the research and design of the prototype. The device is designed and developed in an integrated and modular way, and the synergistic resonance and random vibration are captured separately through different modules. External vibrational excitations are transmitted to the device’s main body, where resonant energy is captured by the P-EMG module, leveraging piezoelectric and electromagnetic induction principles to generate electrical power. Concurrently, random vibrational energy is harnessed by the TENG module, employing the principles of triboelectricity to produce electrical energy.

### 2.2. Device Configuration

**Figure 2:**
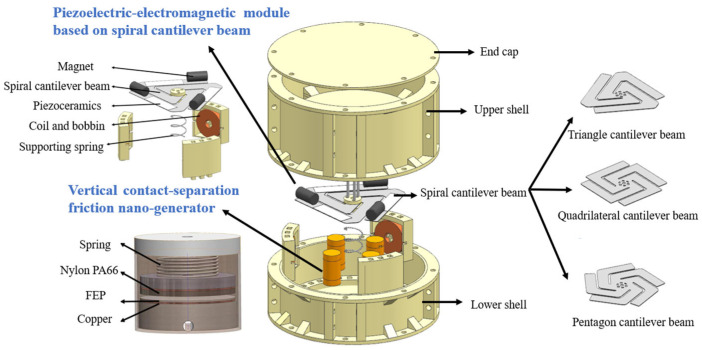
Structural design scheme of piezoelectric–electromagnetic–triboelectric composite power generation device.

Based on the design concept, the structural layout of the device is depicted in Figure 2. The outside of the device is composed of an end cover and disassembled upper and lower shells. The internal unit integrates two power generation modules, of which the core power generation module is a P-EMG module based on a spiral cantilever beam, which is placed in the middle of the whole device. The vibration transmission structure composed of the spiral cantilever beam and the spring plays the role of amplifying the external excitation, causing the module to produce displacement and deformation so as to collect the vibration energy around the resonance frequency. Three types of spiral cantilever beams are designed, namely a triangle cantilever beam (TCB), a quadrilateral cantilever beam (QCB), and pentagon cantilever beam (PCB), to expand the frequency collection range. The beam arm is rotationally symmetrical to ensure that the vibration form of the beam arm is consistent so that the effect of resonance gain is achieved.

The several branch arms on the cantilever beam are rotationally symmetrical, ensuring that the beam maintains vertical vibration consistency and balance during vibration transmission, thereby achieving resonance gain. At the same time, the cantilever beam and device casing have been designed to achieve rapid installation and replacement of three types of cantilever beams under the same casing. When the external vibration environment changes, it can better collect energy in the new environment while ensuring structural stability. The EMG module uses the principle of Faraday’s electromagnetic induction law to change the motion state of the magnet attached to the beam arm through forced vibration to produce displacement, so that the coil cuts the magnetic inductance line to generate electrical energy. The PG module generates electrical energy based on the piezoelectric effect of piezoelectric ceramics. The common piezoelectric ceramics are mainly divided into three categories: barium titanate, lead titanate and lead zirconate titanate [25]. The change in stress caused by external mechanical vibration causes the piezoelectric ceramic to produce a dielectric polarization proportional to the stress, which in turn leads to the internal potential difference, thereby generating electrical energy. The piezoelectric ceramic in this study is PTZ-5 [26]. The auxiliary power generation structure is composed of four identical vertical contact-separation triboelectric nanogenerators, which are installed at the idle position at the bottom of the device. The upper plate material is nylon PA66, the lower plate material is FEP dielectric friction film, and the internal electrodes are continuously separated by spring vibration, resulting in the generation of electric energy, which is transferred by the copper electrodes attached to the two electrodes.

### 2.3. Theoretical Model

It should be noted that, although the piezoelectric, electromagnetic, and triboelectric modules are mechanically coupled in the practical prototype through shared structures such as the cantilever beam and springs, the present theoretical model treats each module as an equivalent single-degree-of-freedom system. This simplified modeling approach is intended to provide first-order insights into the dominant dynamic behavior and parameter influence. The coupling effects, which may introduce additional stiffness and damping interactions, are partially reflected in the experimental results discussed in Section 4.

#### 2.3.1. Establishment of the P-EMG Theoretical Model

**Figure 3:**
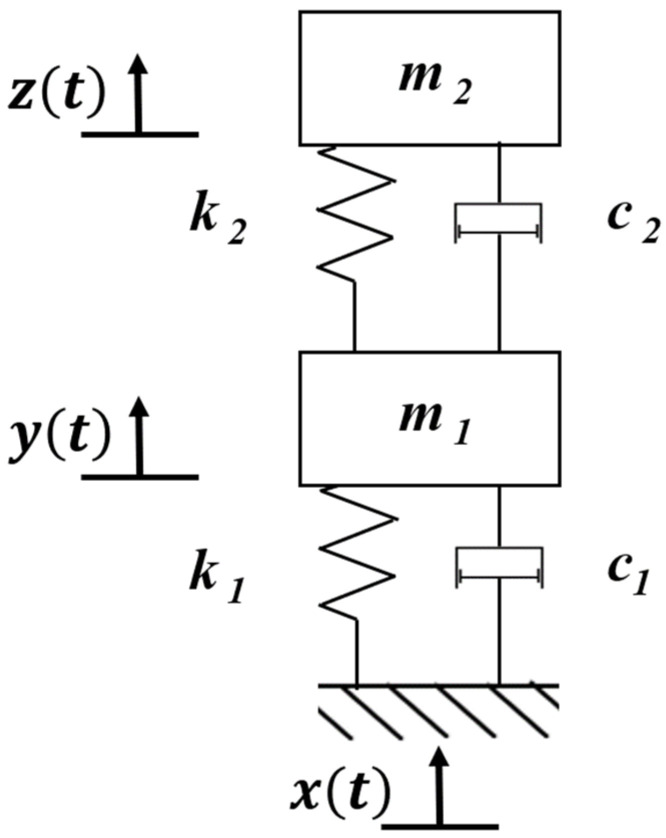
Single-degree-of-freedom system model of spring-mass-damping for P-EMG module.

Due to the brittle characteristic of piezoelectric ceramic materials, in order to avoid fatigue fracture damage caused by overload stress, it is generally attached to the beam-type elastic substrate. The device uses a single-layer pasting method [27] to carry out energy capture, and through the use of a spiral cantilever beam structure, the device is extended as much as possible in a limited space so as to produce a gain vibration effect. The dynamic characteristics of the vibration system are studied by lumped parameter modeling, which is simplified to a spring-mass-damping single-degree-of-freedom system model [28,29,30,31] (Figure 3). In this study, the vibration excitation is modeled as a base excitation. The base displacement is denoted as $x_{b}\left(t\right)$, while y(t) represents the relative displacement of the equivalent mass with respect to the base.

In this formulation, the vibration excitation is described as a base motion. The base displacement is denoted as $x_{b}\left(t\right)$, while $y\left(t\right)$ represents the relative displacement of the equivalent mass with respect to the base. The governing equation of motion for the P-EMG module can therefore be expressed as

(1) $$m\ddot{y}\left(t\right)+c\dot{y}\left(t\right)+ky\left(t\right)=-m{\ddot{x}}_{b}\left(t\right)$$

where m is the equivalent mass of the vibrating system (including the cantilever beam and attached magnet), c is the equivalent viscous damping coefficient (accounting for both mechanical and electromagnetic damping), and k is the equivalent stiffness of the spiral cantilever beam and spring assembly.

Assuming a harmonic base excitation of the form,

(2) $$x_{b}\left(t\right)=X_{b}\sin(\omega t)$$

the steady-state response of the system can be obtained. The amplitude of the relative displacement $y\left(t\right)$ is given by

(3) $$\left|Y\left(\omega \right)\right|=\frac{m\omega^{2}X_{b}}{\sqrt{{\left(k-m\omega^{2}\right)}^{2}+{\left(c\omega \right)}^{2}}}$$

This expression indicates that the vibration response of the P-EMG module is governed by the dynamic stiffness term $k-m\omega^{2}$ and the damping term cω, which is consistent with the standard forced-response solution of an SDOF system under base excitation.

For the piezoelectric generator (PG) module, electrical output is generated through the piezoelectric effect induced by the bending strain of the cantilever beam. The generated voltage is proportional to the mechanical stress acting on the piezoelectric ceramic. Under steady-state vibration, the electrical output power of the piezoelectric module can be approximated as

(4) $$P_{PG}=\frac{U_{PG}^{2}}{R_{PG}}$$

where $U_{PG}$ is the root-mean-square (RMS) output voltage across the resistive load $R_{PG}$.

For the electromagnetic generator (EMG) module, electrical energy is generated based on Faraday’s law of electromagnetic induction. The relative motion between the magnet and coil induces an electromotive force, and the corresponding output power can be expressed as

(5) $$P_{EMG}=\frac{U_{EMG}^{2}}{R_{EMG}}$$

where $U_{EMG}$ is the RMS output voltage and $R_{EMG}$ is the total resistance of the electromagnetic circuit, including both internal and external load resistances.

Accordingly, the total theoretical output power of the P-EMG module can be written as

(6) $$P_{P-EMG}=P_{PG}+P_{EMG}$$

It should be noted that, although the piezoelectric and electromagnetic components are mechanically coupled in the practical prototype, the present theoretical model treats the P-EMG module as an equivalent SDOF system. This simplified model provides first-order insights into the dominant dynamic behavior and parameter influence, while coupling effects are further reflected in the experimental results discussed in Section 4.

#### 2.3.2. Establishment of TENG Theoretical Model

**Figure 4:**
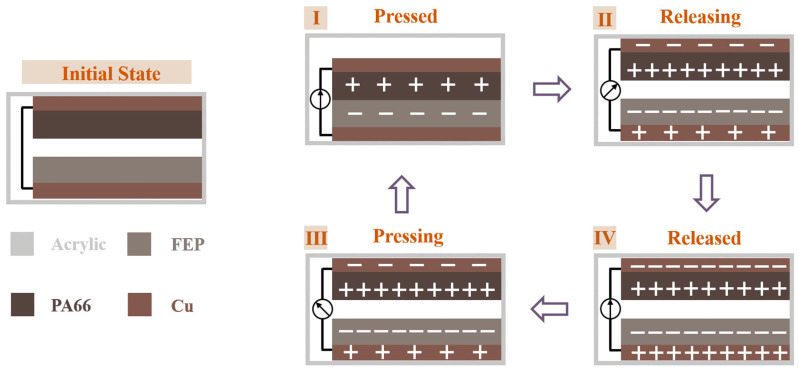
Mechanism of charge generation in contact-separated TENG.

The contact–separation triboelectric nanogenerator is a friction-related energy conversion structure in which electrical output is generated through periodic contact and separation at the material interface. This structure generates electricity in the vertical direction without sliding friction between two plates. This structure can better collect vibration energy in the vertical direction and is more suitable for vibration energy collection devices. The mechanism of charge generation in contact-separated TENG is shown in Figure 4, where two dielectric films are stacked face-to-face to form a friction pair. Under the action of external mechanical forces, the two layers of dielectric material develop a contact-separation motion and the relative distance of their surfaces $u\left(t\right)$ is changed. When two dielectric layers come into contact, the triboelectric effect occurs. According to the triboelectric sequence, the transfer of charge between the two dielectric layers is carried, and the triboelectric charge with the opposite sign of equal magnitude is carried at the same time. Then, the induced potential difference between the two electrodes will be generated, and the path will be formed through the metal electrode plated on the back of the material film to drive the flow of free electrons in the external circuit and convert the vibrational energy into electrical energy.

**Figure 5:**
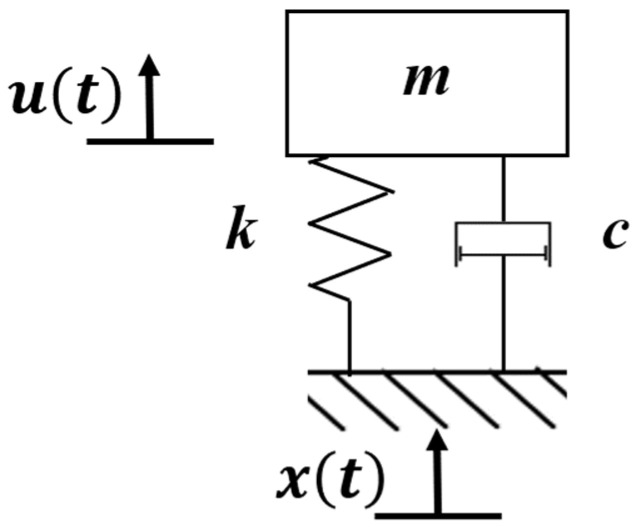
Single-degree-of-freedom system model of spring-mass-damping for friction module.

The dynamic characteristics of the vibration system are studied by lumped parameter modeling, which is simplified to a spring-mass-damping single-degree-of-freedom system model (Figure 5).

Where $u\left(t\right)$ is the vertical relative displacement of the moving substrate, m is the mass of the moving substrate, c is the viscous damping coefficient, and k is the stiffness of the spring, and the equation of motion is

(7) $$m\ddot{u}\left(t\right)+c\dot{u}\left(t\right)+ku\left(t\right)=cA\omega \cos\left(\omega t\right)+kAsin\left(\omega t\right)$$

Solving the same calculation method as the P-EMG system above, the simplest solution of the displacement of the triboelectric power generation system can be obtained as follows:

(8) $$u\left(t\right)=\sqrt{{B_{1}}^{2}+{B_{2}}^{2}}\sin(\omega t+\tan^{-1}\frac{B_{1}}{B_{2}})$$

$$\left\{\begin{array}{c} B_{1}=\frac{-m\omega^{3}cA}{{\left(k-m\omega^{3}\right)}^{2}-{\left(c\omega \right)}^{2}}\\ B_{2}=\frac{A\left(k^{2}-km\omega -c^{2}\omega^{2}\right)}{{\left(k-m\omega^{3}\right)}^{2}-{\left(c\omega \right)}^{2}} \end{array}\right.$$

From the literature [32], it can be seen that the analytical expressions of the contact separated triboelectric power generation current and voltage are

(9) $$\begin{array}{rl} I(t)=-\frac{\sigma d_{0}}{R\varepsilon_{0}}+ & \frac{\sigma \left(d_{0}+u(t)\right)}{R\varepsilon_{0}}\exp\left[-\frac{1}{RS\varepsilon_{0}}\left(d_{0}t+\int_{0}^{t}u(z)dz\right)\right]\\ & +\frac{\sigma d_{0}}{R\varepsilon_{0}}\frac{d_{0}+u(t)}{RS\varepsilon_{0}}\int_{0}^{t}\exp\left[\frac{1}{RS\varepsilon_{0}}\left(d_{0}(z-t)+\int_{t}^{z}u(r)dr\right)\right]dz \end{array}$$

(10) $$\begin{array}{rl} V(t)=-\frac{\sigma d_{0}}{\varepsilon_{0}}+ & \frac{\sigma \left(d_{0}+u(t)\right)}{\varepsilon_{0}}\exp\left[-\frac{1}{RS\varepsilon_{0}}\left(d_{0}t+\int_{0}^{t}u(z)dz\right)\right]\\ & +\frac{\sigma d_{0}}{\varepsilon_{0}}\frac{d_{0}+u(t)}{RS\varepsilon_{0}}\int_{0}^{t}\exp\left[\frac{1}{RS\varepsilon_{0}}\left(d_{0}(z-t)+\int_{t}^{z}u(r)dr\right)\right]dz \end{array}$$

where $\varepsilon_{0}$ is the vacuum dielectric constant, σ is the surface charge density of the friction pair, $d_{0}$ is the effective thickness constant, S is the area of the dielectric film, and R is the load resistance.

## 3. System Modeling

To clarify the applicability of the theoretical analysis, we note that although the three modules are mechanically coupled in the prototype through shared structures (e.g., the cantilever beam and springs), each module is modeled as an equivalent single-degree-of-freedom system to provide first-order insights into dominant dynamics and parameter effects. Coupling-induced stiffness and damping interactions are discussed with reference to the experimental results in Section 4.

### 3.1. Simulation Setup

All simulations were performed using COMSOL Multiphysics. The cantilever beam was modeled with a fixed boundary condition at the clamped end, while the remaining parts were allowed to vibrate freely. Material properties, including density, Young’s modulus, and piezoelectric constants, were assigned according to manufacturer datasheets and literature values.

A linear viscous damping model was adopted in the simulations to represent structural damping. Mesh independence was verified by refining the mesh until variations in displacement and stress results became negligible.

For the electromagnetic module, the geometric parameters of the magnet and coil, as well as the magnetic material properties, were explicitly defined in the model.

### 3.2. Simulation Analysis of Cantilever Beam Force Displacement

In order to prolong the service life of the device and avoid the fatigue crack or static load fracture of the brittle cantilever beam, the stress of the spiral cantilever beam is first simulated and analyzed as shown in Figure 5. A force of 5 N is applied to the free end of the projection length of 9.6 cm and thickness of 0.4 mm, and the feasibility of the structural design is verified by comparing the maximum surface stress with the maximum load that the material can bear. The simulation results show that the maximum load of the beam is 3.56 × 103, which does not exceed the maximum load of the 304 stainless steel material used. It is indicated that the spiral beam structure can effectively uniform the surface stress value and alleviate the stress concentration phenomenon, and the structural design is feasible.

The vibration will cause the displacement of the spiral cantilever beam in the vertical direction. The PG module realizes the generator mechanism through the force of the piezoelectric ceramic in the vertical direction of the beam, and the EMG module relies on the vertical swing of the end of the beam to make the coil cut the magnetic inductance line to generate electric energy. It is obvious that the displacement of the cantilever beam in the vertical direction determines the power generation effect. In this study, the ratio of the unit force applied at the end of the beam to the maximum displacement of the beam in the vertical direction is defined as the equivalent stiffness, which is used to measure the response of cantilever beams of different structures to vibration excitation forces. The results show that the displacement range of the TCB is 0–0.0198 m, the displacement range of the QCB is 0–0.0088 m, and the displacement range of the PCB is 0–0.0044 m. Three types of beam structures with different equivalent stiffness (Table 1) broaden the range of applicable working conditions of the device.

The reported values are presented as mean values. The measurement uncertainties primarily stem from the accuracy of the measurement devices used for stiffness determination. The uncertainty in the equivalent stiffness values is ±5% due to measurement inaccuracies in the applied force and displacement measurement.

### 3.3. Simulation Analysis of P-EMG Model

**Figure 6:**
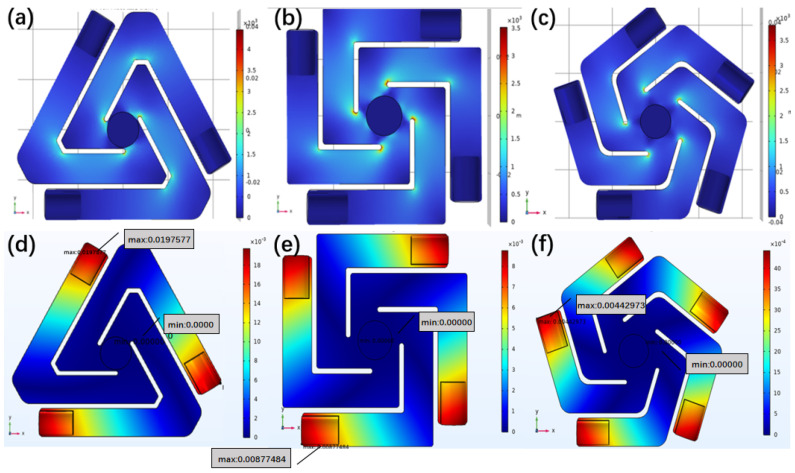
Force simulation diagram of TCB (**a**), QCB (**b**) and PCB (**c**) and displacement simulation diagram of TCB (**d**), QCB (**e**) and PCB (**f**).

In order to investigate the influence of structural parameters of magnets and coils on power generation efficiency, this study used COMSOL simulation software and TCB as the structural carrier. The common surface vibration loads of low-speed marine diesel engines, 5 N and 20 Hz, were used as reference conditions to explore the influence of parameters of magnets and coils on power generation efficiency. Taking the TCB configuration as an example, the distribution of displacement and stress of the device is explored by applying the excitation force of 5 N to the device. The excitation of the device is transmitted through the beam to the piezoelectric ceramic, and the piezoelectric effect generates electrical energy. The surface voltage of piezoelectric ceramics and their output voltage waveforms are obtained. It can be seen from the surface pressure distribution (Figure 6a) of the piezoelectric ceramic that under the action of the sinusoidal excitation force, the potential of the edge end of the material changes greatly, and the potential difference between the two ends of the piezoelectric ceramic is obvious. The waveform after stabilization is shown in Figure 6b, where the maximum voltage reaches 1.6 V, which shows the positive result of harvesting.

Minor waveform distortion relative to an ideal sinusoidal signal is observed, which can be attributed to mechanical nonlinearity and electrical loading effects. The total harmonic distortion is approximately 3–5%.

According to the law of electromagnetic induction, the induced electromotive force of a coil in a magnetic field can be expressed as

(11) $$U=-Blu\times 10^{-4}\;\left(V\right)$$

where *B* is the magnetic field strength, *l* is the effective length of the coil in the magnetic field, and *u* is the relative velocity of the coil in the magnetic field.

**Figure 7:**
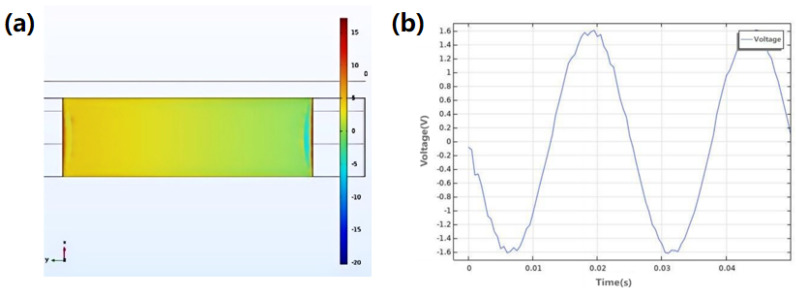
Surface pressure distribution of piezoelectric ceramics (**a**) and output voltage of piezoelectric ceramics (**b**).

**Figure 8:**
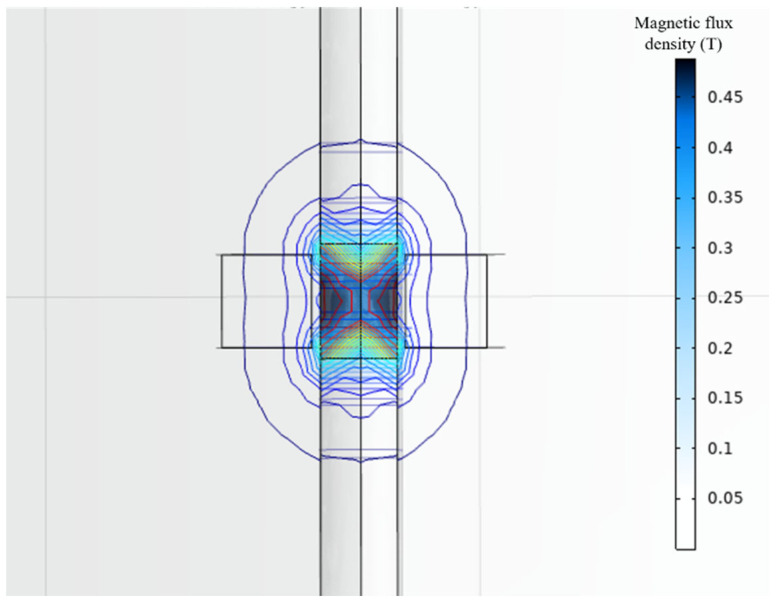
Simulation of magnetic field distribution of magnets.

**Figure 9:**
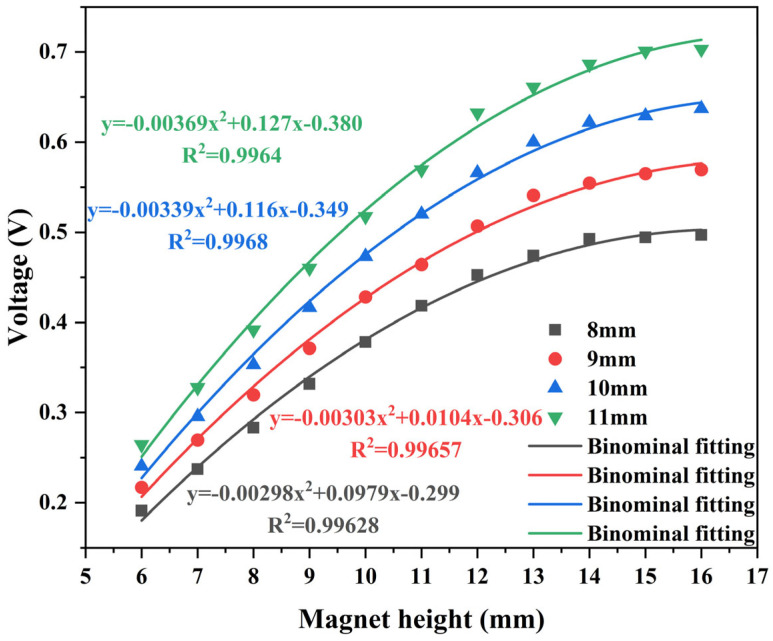
Relationship between magnet diameter, height, and voltage.

As can be seen from the above equation, the voltage of the electromagnetic module mainly depends on three aspects: First is the magnetic field strength, which depends on the structural parameters of the magnet itself. The second is the effective length of the coil, which depends on the ratio of the width to height of the coil. The third factor is relative velocity, which depends on the magnitude of the vibration excitation for the vibration energy harvesting device. Therefore, for electromagnetic modules, the diameter and height of the magnet, as well as the width-to-height ratio of the coil, are important research parameters. The magnetic field distribution of the magnet was simulated and analyzed using COMSOL software. The simulation pattern of magnetic field distribution of the magnet is shown in Figure 7. Magnets exhibit a two-level distribution in the vertical direction, and when the coil is wrapped around the magnet, it can effectively cut the magnetic induction line under relative motion. Based on the simulation model, the parameters of the magnet and coil in the electromagnetic power generation module were explored to obtain the optimal parameters, and the results are shown in Figure 8 and Figure 9. Under the condition that variables such as the control magnet material and the number of coil turns are consistent, the influence of magnet diameter and height on the power generation effect is positively correlated, which tends to be flat after the height reaches 12 mm. The influence of the ratio of magnet to coil width and height on the power generation effect first increases and then decreases, reaching peaks of 0.52 V and 0.49 V at the ratios of about 0.6 and 0.8, respectively. Based on the simulation results and the limitations of the actual processing conditions, the magnet diameter is 10 mm, the height is 12 mm, the aspect ratio is 0.83, the number of turns of the coil is 2400 turns, the diameter difference is 14 mm, and the diameter difference-to-height ratio is 0.5. These parameters laid the foundation for the success of the experiment.

Symbols denote experimental data, and the curves show second-order polynomial regression for trend guidance (not a theoretical model).

Symbols represent experimental data points, while the solid curves indicate third-order polynomial regression fits used only to guide the trend (not a theoretical model).

## 4. Experimental and Discussion

### 4.1. Fabrication and Assembly of the Device

**Figure 10:**
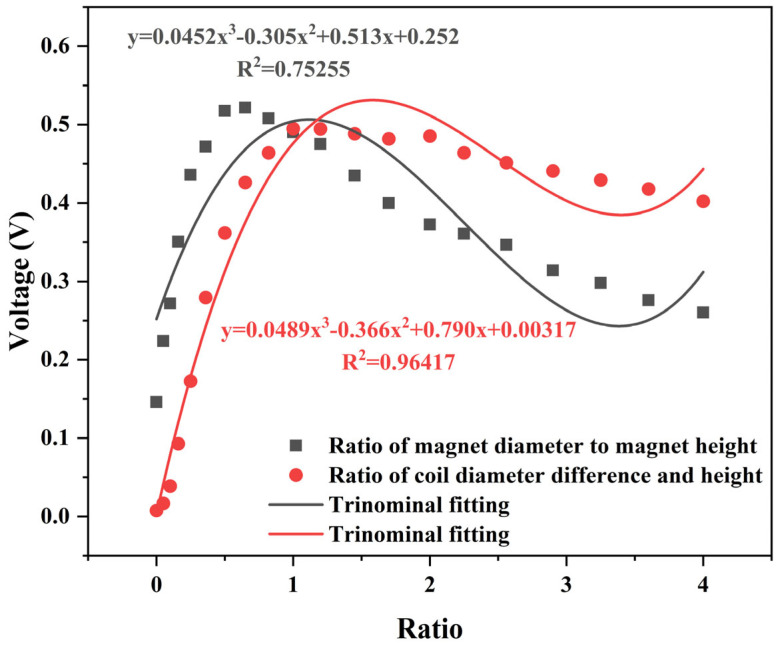
Relationship between magnet and coil aspect ratio and voltage.

Based on the optimal parameters of the previous research, 3D printing and CNC machining technologies were used to prepare various parts of the PET-HVEH. The device was fabricated through welding, pasting and bolting processes to ensure that its structure was firm and met the design requirements (Figure 10). In order to verify the effectiveness and reliability of the research, the PET-HVEH was tested and analyzed by using the laboratory test device, which included key equipment such as a signal generator, power amplifier, exciter and oscilloscope (Figure 10). The combination of these devices not only meets the needs of the experiment but also ensures that the performance of the device is fully monitored during the experiment. The empirical examinations stand as a pivotal facet within this investigative endeavor, encompassing a methodical evaluation and analysis predicated upon the operational dynamics of the apparatus. This undertaking seeks to endow the research with veritable data substantiation, thereby serving as an enduring bedrock for prospective refinements and optimizations in the architectural design of the device. Such meticulous scrutiny is indispensable to safeguard the scientific integrity and veracity of the research outcomes.

### 4.2. Energy Efficiency Analysis

**Figure 11:**
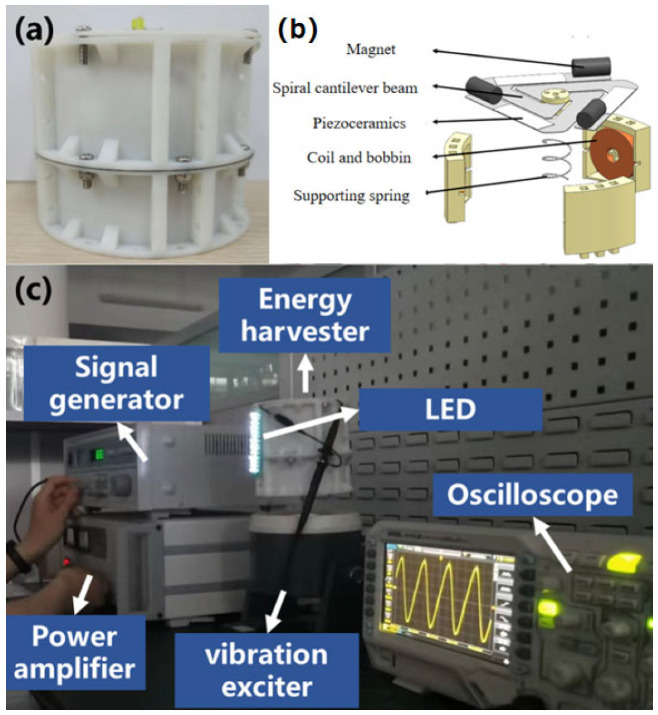
(**a**) Physical device, (**b**) exploded view of the experimental setup, and (**c**) laboratory test equipment.

Initially, efficiency parameters of the apparatus are measured through a series of assessments. Acceleration sensors are employed to quantify the vibrational acceleration induced by the exciter input. Concurrently, an electronic scale is utilized to determine the mass of the device, while a multimeter gauges the resistances across various components. In order to determine the voltage output characteristics of each power generation module, an oscilloscope was used to monitor the output electrical signals of each power generation module to record the maximum value of output voltage and other data. The oscilloscope displays a generation waveform, as shown in Figure 11. From the figure, it can be seen that under the condition of sinusoidal vibration excitation, all three vibration power generation modules successfully output voltage. It is worth noting that the voltage output by the piezoelectric module and electromagnetic module is in the shape of a sine function, while the friction nanogenerator shows a periodic and jumping voltage shape. This may be due to the internal structural characteristics of the friction nanogenerator, where the two plates continuously contact and separate, causing the voltage to show a step mutation at the moment of separation.

To ensure the reliability of the reported output power, electrical measurements were determined under clearly defined loading conditions. During the experiments, each power generation module was connected to a resistive load, and the voltage across the load was measured using an oscilloscope. The corresponding output current was obtained based on the measured voltage and the known load resistance. No additional rectification or signal-conditioning circuits were applied unless otherwise specified.

According to the above measurement parameters, the energy conversion efficiency can be determined as shown in Equation (12), and the measurement parameters and the symbolic interpretation of each parameter are shown in Table 2. By measuring the power of the device at the resonance frequency of 23 Hz and the maximum acceleration of 0.8 m/s^2^, the maximum output power of the device reaches 2.86 mW under a sinusoidal excitation at 23 Hz with an acceleration amplitude of 0.8 m/s^2^, and the maximum averaged electrical output power of the device reaches 2.86 mW across the optimal resistive load. Here, the reported output power refers to the averaged electrical power over one steady-state vibration period rather than the instantaneous peak power.

In this work, the energy conversion efficiency is defined as the ratio of the averaged electrical output power to the input mechanical power:

(12) $$\eta =\frac{P_{elec}}{P_{mech,in}}$$

The input mechanical power is estimated based on the measured vibration acceleration of the shaker and the effective moving mass of the device, assuming steady-state sinusoidal excitation. For example, at a vibration frequency of 23 Hz and an acceleration amplitude of 0.8 m/s^2^, the calculated input mechanical power and the measured electrical output power result in a maximum conversion efficiency of 36.81%. The uncertainty of the calculated efficiency mainly arises from the acceleration sensor accuracy and electrical measurement errors.

(13) η=πU1I1+U22R2+U32R3A2ω3−ω2m+k

**Figure 12:**
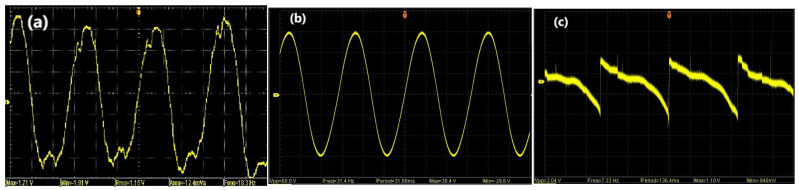
Representative output voltage waveforms of the piezoelectric (**a**), electromagnetic (**b**), and triboelectric (**c**) modules under sinusoidal vibration excitation at 23 Hz and 0.8 m/s^2^.

The averaged output power was calculated over one steady-state vibration period based on the measured voltage signal across the resistive load. The simulation results are summarized in Figure 12.

The reported values are presented as mean values. The measurement uncertainties primarily stem from the accuracy of the measurement devices used for parameter determination. The uncertainty in the acceleration amplitude is ±2%, frequency is ±1%, and resistance is ±3%.

**Figure 13:**
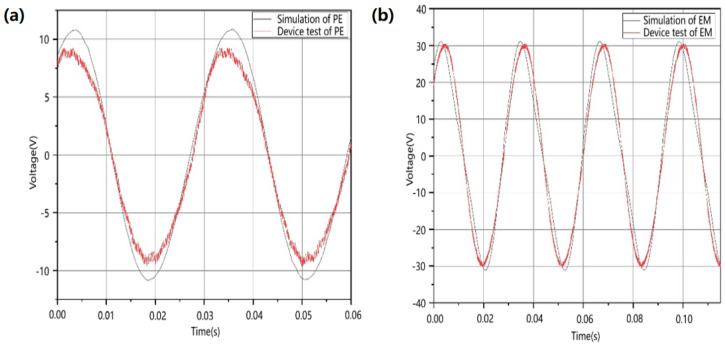
Piezoelectric module simulation and physical comparison test. (**a**) Electromagnetic module simulation and physical comparison test (**b**).

The P-EMG module was tested and analyzed independently. Comparative testing was conducted on the piezoelectric module and electromagnetic module, subjecting them to simulation conditions with identical parameters. The outcomes revealed a commendable concordance between the experimental and simulated result curves, as shown in Figure 13. This alignment distinctly underscores the effectiveness and feasibility of the simulation experiments, simultaneously affirming the accuracy and reliability of the physical testing for both modules. These findings reflect a high level of output efficiency.

**Figure 14:**
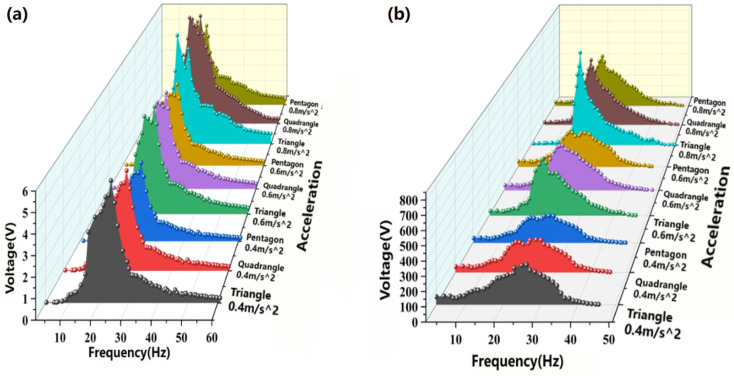
Sweep test of piezoelectric module (**a**) and electromagnetic module (**b**).

The P-EMG module based on a spiral cantilever beam was swept to explore the response of piezoelectric ceramics and electromagnetic structures under different beam structures, accelerations and frequencies. As shown in Figure 14, both the piezoelectric module and the electromagnetic module reach their peak at a vibration frequency of about 22 Hz, and as the frequency increases, the output voltage first shows an increasing trend and then a decreasing one. It is worth noting that under the same vibration acceleration excitation conditions, whether it is the piezoelectric module or the electromagnetic module, the output voltage size is always triangle > quadrilateral > pentagon, which is consistent with the trend of stiffness changes in the three types of beams. Under the same beam structure, as the acceleration increases, the generated voltage also increases. From a practical perspective, the vibration collection frequency domain is judged based on the lowest input voltage at which the device can successfully collect energy, with the lowest voltage of 2.7 V for the PG module and 300 mV for the EMG module. The bandwidth of the TCB structure can reach 16.4 Hz, the width of the QCB is 15.8 Hz, and the bandwidth of the PCB structure is 14.4 Hz, which proves that the structure can effectively broaden the vibration frequency domain and improve the power generation capacity.

Minor waveform distortion relative to an ideal sinusoidal signal is observed during the excitation process. The total harmonic distortion is approximately 3–5%, indicating a small deviation from ideal harmonic excitation.

**Figure 15:**
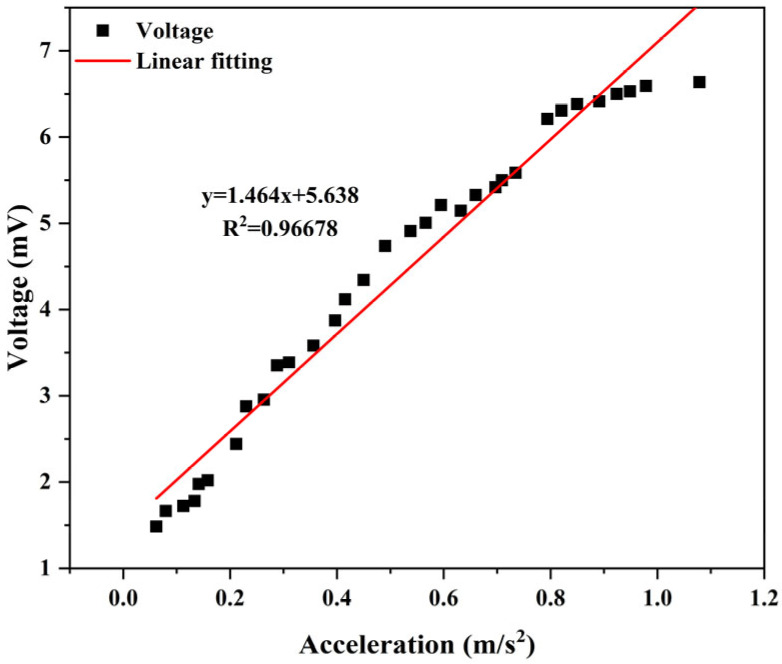
Relationship between the output voltage of the TENG module and the vibration acceleration.

The power generation structure of the TENG module is not affected by the vibration frequency but only depends on the vibration acceleration. The experimental analysis in Figure 15 shows that the output voltage of the friction module is approximately positively correlated with the vibration acceleration, and the resulting voltage is approximately linear upward as the acceleration given to the friction component increases. Outside the resonance region, the output voltage rapidly decreases and is therefore not shown for clarity.

The approximately linear voltage–acceleration relationship also indicates stable contact–separation behavior at the triboelectric interface, which is beneficial for friction-related energy conversion applications. Linear regression was performed on the voltage–acceleration data, yielding a coefficient of determination (R^2^) of 0.967, indicating good linearity within the tested acceleration range.

Table 3 shows the vibration energy harvesters (VEHs) developed by some of the research institutes, comparing parameters such as power generation mode, device structure, type of captured vibration, and output power. The table shows that most of these devices adopt a beam structure and use a single vibration form for composite power generation, and different devices have produced relatively positive effects.

Table 4 shows the improvement in the main technical indicators, such as the maximum output power, the maximum bandwidth and the maximum conversion efficiency of the device, which proves the feasibility of applying the device in the field of vibration energy harvesting. Table 4 presents the main technical indicators of the maximum output power, maximum vibration collection frequency domain, and maximum conversion efficiency of this device. Compared with the vibration energy collection devices developed by various scholars, as shown in Table 3, this design has higher power generation and wider bandwidth, proving the feasibility of this device in vibration energy collection applications.

It should be noted that the output power and bandwidth values reported in Table 3 and Table 4 were obtained under different excitation conditions and device dimensions in the referenced studies. Therefore, the comparison presented here is intended to provide a qualitative performance reference rather than a strictly normalized quantitative evaluation. The sensitivity value is provided for reference. Furthermore, the proposed device is primarily designed as an energy harvester rather than a calibrated acceleration sensor.

The reported values are presented as mean values. The measurement uncertainties mainly arise from the accuracy of the measurement devices used for output power, bandwidth, and efficiency determination. The uncertainty in output power is ±2%, bandwidth is ±0.5 Hz, and conversion efficiency is ±1%.

The present study focuses on the mechanical-to-electrical energy conversion performance of the proposed device. The operation of additional electronics, such as signal conditioning, filtering, and wireless transmission modules, is beyond the scope of this work and will be investigated in future studies. Moreover, the influence of multi-axis vibrations on the device performance is expected to be limited due to the dominant sensitivity along the primary vibration axis.

## 5. Conclusions

The main conclusions of this study can be summarized as follows:

1. A low-frequency hybrid vibration energy harvester based on a spiral cantilever beam was successfully designed and fabricated.
2. The proposed PET-HVEH demonstrates broadened operating bandwidth and enhanced output power under low-frequency vibration excitation.
3. A maximum averaged output power of 2.86 mW and an energy conversion efficiency of 36.81% were achieved at 23 Hz.
4. The experimental results confirm the feasibility and effectiveness of combining piezoelectric, electromagnetic, and triboelectric mechanisms.

## Figures and Tables

**Table 1:** Equivalent stiffness of three types of spiral cantilever beams.

Structure	Equivalent Stiffness (N/m)	Measurement Uncertainty
Triangle cantilever beam	253.07	±5%
Quadrilateral cantilever beam	569.81	±5%
Pentagon cantilever beam	1128.74	±5%

**Table 2:** Parameter symbol definitions.

Name	Value	Name	Description
Acceleration amplitude A (m/s^2^)	0.3–0.9	U1	Piezoelectric ceramic voltage (V)
Mass m (g)	580	U2	Electromagnetic module voltage (V)
Resonant frequency f_n_ (Hz)	23	U3	Triboelectric module voltage (V)
Equivalent stiffness k (N/m)	1 × 10^7^	I1	Piezoelectric ceramic current (A)
Piezoelectric ceramic load resistance R_1_ (Ω)	1 × 10^3^	I2	Electromagnetic module current (A)
Coil resistance R_2_ (Ω)	54.2	I3	Triboelectric module current (A)
Triboelectric module resistance R_3_ (kΩ)	400	ω	Characterize vibration frequency (rad/s)

**Table 3:** Comparison of vibration energy harvester.

Power Generation Type	Structure Type	Energy Acquisition Mode	Research Department	Maximum Power	Effective Frequency Domain
Piezoelectric [33]	Straight cantilever beam structure	Resonance	Tianjin University	0.01 mW	5.0 Hz
Piezoelectric–electromagnetic [34]	Spiral beam fixed at both ends	Resonance	Harbin Institute of Technology	2.21 mW	3.5 Hz
Piezoelectric–electromagnetic [35]	Straight cantilever beam structure	Resonance	Kwangwoon University	1.97 mW	5.0 Hz
Triboelectric–electromagnetic [36]	Piston structure	Random vibration	Universitetet i Bergen	0.50 mW	3.0 Hz
Triboelectric–piezoelectric–electromagnetic [37]	Fixed support at both ends	Resonance and random vibration	Chongqing University	0.41 mW	9.6 Hz

**Table 4:** Comparison of output parameters.

Name	Value	The Percentage of Improvement
Maximum output power	2.86 mW	29.41% ± 2%
Maximum bandwidth	16.4 Hz	70.83% ± 0.5 Hz
Maximum conversion efficiency	36.81% ± 1%	

## Data Availability

All data supporting the findings of this study are available within the article.
